# Early intervention program for psychosis in a portuguese hospital: The first results of the open program

**DOI:** 10.1192/j.eurpsy.2021.1421

**Published:** 2021-08-13

**Authors:** H. Simião, L. Santana, R. Silva, J. Gago

**Affiliations:** Psychiatry, Centro Hospitalar de Lisboa Ocidental, Lisbon, Portugal

**Keywords:** case manager, individual care plan

## Abstract

**Introduction:**

OPEN is a structured intervention program for patients who present with untreated psychosis at an early stage, under the guidance of a case manager and a periodic evaluation performed by a multidisciplinary team.

**Objectives:**

The aim of the OPEN program is to create an individual care plan, promoting recovery, functionality, quality of life and prevent relapses. One year after implementation of the program, we present the first results.

**Methods:**

We performed a retrospective review of the patients’ clinical profiles included in the OPEN program.

**Results:**

Nine patients were included in the program. The most frequent diagnosis was schizophrenia (n=5). By the time of inclusion in the program, 5 patients used cannabis frequently; one patient kept substance use. At the 6^th^ month assessment, 4 in 5 patients showed an improvement in social functioning (Personal and Social Performance Scale; ± 16 points), and a decrease in symptom severity in all (Brief Psychiatric Rating Scale; ± 11 points). Due to COVID-19 pandemics, group interventions were suspended, and some visits were performed by teleconsultation. No patients lost follow-up.

**Conclusions:**

We observed an overall positive result of the first months of this program, regarding both functionality and clinical outcomes. The main obstacle so far is the impossibility of performing group interventions since the start of the contingency measures regarding COVID-19 pandemics. We expect further results of the OPEN program with the inclusion of more patients.

